# Possibilities for secondary data use of electronic health records with WiseSpace de-identification

**DOI:** 10.3389/fmed.2025.1639342

**Published:** 2025-08-29

**Authors:** Olga Vovk, Ali Ghasempour, Gunnar Piho, Peeter Ross

**Affiliations:** ^1^Department of Software Science, Tallinn University of Technology, Tallinn, Estonia; ^2^IT College, Tallinn University of Technology, Tallinn, Estonia; ^3^Department of Health Technologies, Tallinn University of Technology, Tallinn, Estonia

**Keywords:** de-identification, anonymization, pseudonymization, electronic health records (EHRs), secondary use, European healthdata space (EHDS), general data protection regulation (GDPR)

## Abstract

**Introduction:**

The secondary use of Electronic Health Records (EHRs) holds significant potential for advancing research, public health, and innovation. However, data sharing is often limited by privacy regulations, requirements, and technical complexity. This study introduces Design Science (DS) research on the evidence-based design of WiseSpace—a tool specifically tailored to address these challenges by enabling the secure, regulation-compliant de-identification of healthcare data, particularly for non-technical users.

**Methods:**

The research utilizes DS methodology to develop and evaluate the de-identification solution. This approach includes problem investigation through literature review, existing method and tool evaluation, and expert interviews; treatment design based on the identified challenges; treatment validation; and treatment implementation.

**Results:**

WiseSpace provides tools for personal, identifiable health data detection, de-identification, and re-identification as well as risk assessment. The tool supports common health data standards and its intuitive user interface allows healthcare professionals, individuals, and researchers to perform data management-related tasks without requiring technical expertise.

**Discussion:**

WiseSpace addresses critical gaps in existing anonymization solutions by providing domain-specific support for healthcare data and ensuring compliance with the General Data Protection Regulation (GDPR) and the European Health Data Space (EHDS). It offers automation and risk mitigation solutions and simplifies workflow, enabling secondary data use. Use cases demonstrate the solution's utility for organizations and individuals.

## 1 Introduction

In the evolving digital healthcare space, EHRs play a critical role not only in delivering personalized care but also in enabling secondary data use for research, public health improvement, policy-making, and innovation. EHRs are valuable resource for understanding health trends, statistics, and treatment efficiency as well as building analytical tools.

Healthcare data use can be divided into two main types: primary use and secondary use. Primary data use includes patient care and healthcare operations (1) or, more specifically, delivering care to the individual from whom the data were collected (2). Secondary data use (or reuse) occurs when health data are used outside of direct healthcare delivery. According to Safran et al. (2), this includes analysis, research, quality and safety measurement, public health, payment, provider certification, marketing, business applications, and other commercial activities.

Moreover, the EHDS defines the primary use of electronic health data as the processing of personal data for the provision of health services to assess, maintain, or restore the health of the individual. This includes the prescription, dispensation, and provision of medicinal products and medical devices as well as the related social security, administrative, or reimbursement services (3).

Secondary use refers to the processing of electronic health data for purposes beyond individual care. These purposes include public interest activities in public and occupational health, supporting public authorities and regulatory bodies, producing official statistics, education and teaching, scientific research, the innovation and development of health-related products and services, algorithm training and validation, including AI systems and digital health applications, and the provision of personalized healthcare based on population-level data. Secondary use may involve data originally collected for primary use or data gathered specifically for secondary purposes (3).

Secondary data use brings multiple benefits, such as improving healthcare delivery and reducing costs. Data have great potential for secondary use in research, business decisions, and product development. However, despite the value of this data, sharing is often limited by privacy laws and technical barriers (4). Various studies show significant challenges in the process of data sharing and secondary use. These include understanding the data and preparation steps (5), data quality challenges (6), cybersecurity challenges (7), privacy concerns, and data ownership (8).

The European Union has established a robust regulatory framework for personal data protection and data sharing, including GDPR and EHDS regulations. These regulations emphasize the protection of individuals' health data while empowering secure reuse for societal benefit. However, in practice, healthcare providers and other organizations face significant barriers in complying with these regulations. Another important regulation is the US Health Insurance Portability and Accountability Act (HIPAA). HIPAA sets the standards for managing, transmitting, and storing protected health information and compliance therewith is mandatory for healthcare providers, insurers, and other organizations handling patient data (9). Even though HIPAA is US law, it has an impact on global health data management practices.

One of the key approaches for secure data sharing for secondary use is personal data de-identification, which uses techniques such as anonymization and pseudonymization (10). Article 4 of the GDPR defines “personal data” as “any information relating to an identified or identifiable natural person (‘data subject'); an identifiable natural person is one who can be identified, directly or indirectly, in particular by reference to an identifier such as a name, an identification number, location data, an online identifier or to one or more factors specific to the physical, physiological, genetic, mental, economic, cultural or social identity of that natural person” (11). Unlike the GDPR, which provides a broad description, HIPAA specifies 18 identifiers that are considered personally identifiable information. These identifiers include name, address, dates related to an individual (including date of birth, admission date, discharge date, date of death), phone number, email, social security number, medical record number, and others (12). According to the Safe Harbor Provision, these identifiers must be removed from a dataset for it to be considered anonymized.

However, the effective implementation of de-identification requires specialized knowledge in the healthcare field, an understanding of data privacy regulations, and technical skills. This research proposes a more comprehensive solution than simply data de-identification. We propose and evaluate a framework for secure data sharing that involves multiple stakeholders. Healthcare organizations can benefit from this solution as it simplifies the data de-identification process, making it faster and more efficient. Individuals can de-identify their own data for personal benefit, such as using AI tools for analytics or donating data to research institutions, thus benefiting society. Research institutions can collect de-identified data for research purposes shared by individuals or healthcare organizations.

## 2 Methods

**Figure 1:**
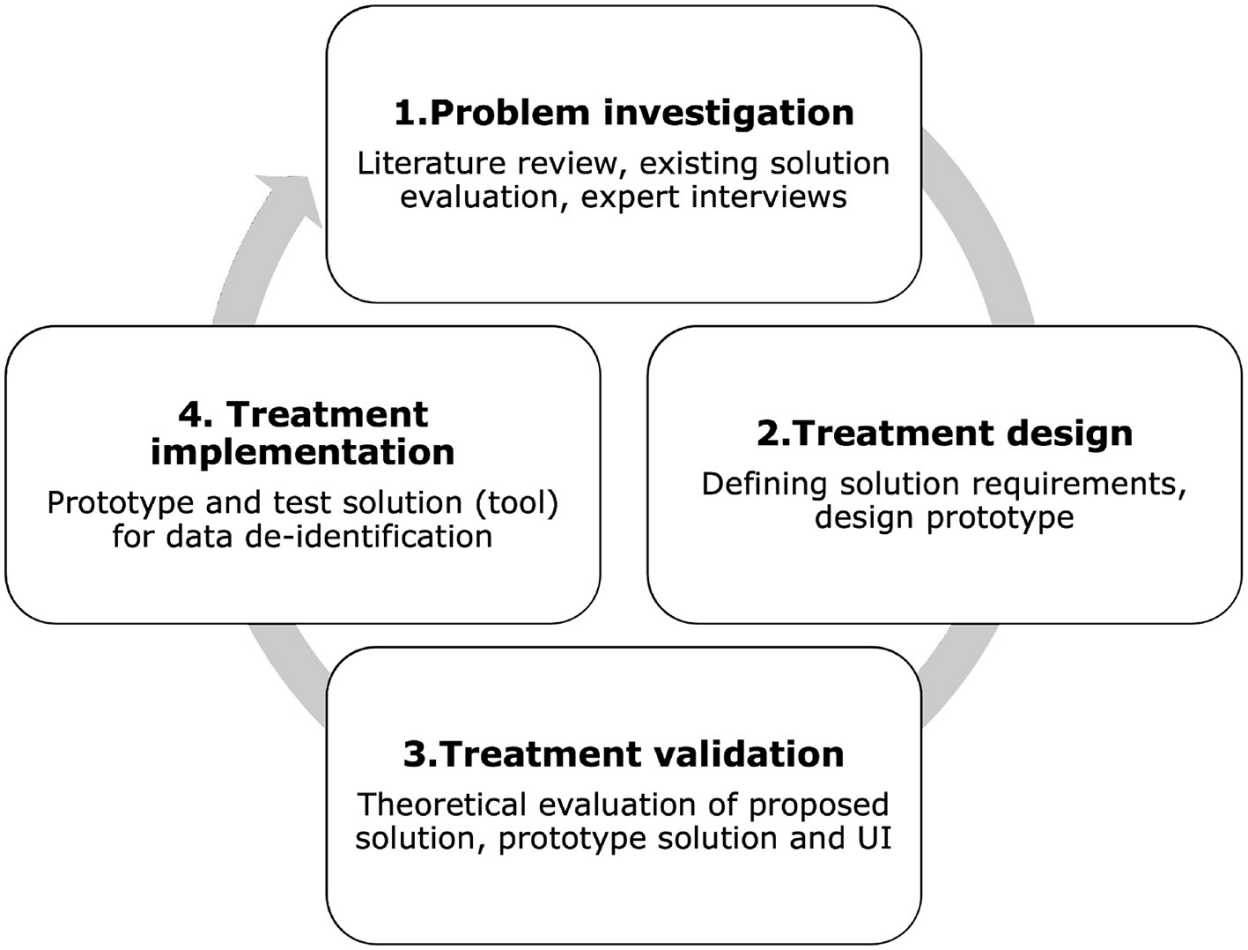
Applied design science research methodology.

We used the Design Science (DS) methodology (13) to develop and evaluate the proposed tool. DS methodology consists of the following phases: (1) problem investigation; (2) treatment design; (3) treatment validation. In this context, the term “treatment” is used instead of “solution” to distinguish the possibility that the artifact may solve a problem only partially, not solve it at all, or even introduce a new problem (13). By using the term “treatment,” similarly to the healthcare field, we specify that the artifact interacts with the problem context to treat a real-world problem. For this reason, it is not limited to designing artifacts but creates the desired interaction between the artifact and the problem context (13). Although DS methodology formally uses the term “treatment,” in this article we also use the term “solution” for clarity, especially when describing practical aspects of the artifact. Both terms are used interchangeably without altering their meaning. Treatment implementation is not part of DS, but it is part of the engineering cycle; we included this step to demonstrate the role of the prototype in our research. Figure 1 shows the DS methodology used in this research. The following steps expand on the methodology:

**Step 1**. Problem investigation includes literature review, existing tool evaluation, and interviews with experts.

**Step 2**. Treatment design involves defining the solution requirements and designing the prototype based on the problems identified in Step 1.

**Step 3**. Treatment validation evaluates how our solution contributes to stakeholders goals.

**Step 4**. Treatment implementation is not part of DS but is part of the engineering cycle. This phase extends the research to real problem application. In this phase, we design and test the tool prototype for data de-identification and risk assessment.

### 2.1 Problem investigation

For problem investigation, we employed a combination of literature review, current tool analysis, and interviews with experts.

#### 2.1.1 Literature review

Multiple researchers address the question of health data anonymization in their work. Among them are Gkoulalas-Divanis et al. (14), who published a survey of existing algorithms, Jayabalan et al. (15), who gave a general overview of data anonymization techniques and Pawar et al. (16), who presented various anonymization techniques with a focus on comparing existing algorithms. Moreover, anonymization in healthcare is an actively developed and investigated field. An example of this is the recent research by Olatunji et al. (17), which reviews anonymization tools and models, and the research of Shamsinejad et al. (18), which is dedicated to anonymization algorithms and methods in Big Data. The literature review shows that there are several ways to approach the problem of data de-identification and sharing. Various methods and techniques are available; however, they all have advantages and disadvantages. For an unexperienced user without a deep technical background and understanding of privacy rules, selecting and applying these methods and techniques to real data is quite challenging.

#### 2.1.2 Existing tool evaluation

Our research shows that existing tools primarily focus on anonymizing general data and are not equipped to handle the specific formats and standards used in healthcare (19). In this research, we evaluated the anonymization tools ARX (20), sdcMicro (21), μ-ANT (22), Amnesia (23), and Anonimatron (24). Based on our results, ARX is the most suitable tool that has comprehensive documentation, a graphical user interface (UI), and various functionalities. However, to use this tool, users must be familiar with privacy and risk models and methods of data anonymization to apply appropriate settings to a dataset. sdcMicro also has many benefits, including a risk calculation functionality. Nevertheless, the tool requires knowledge of R-language and mathematics, which is challenging to unexperienced users. μ-ANT also requires technical knowledge and is missing the risk assessment functionality. Amnesia is a flexible anonymization tool with a graphical user interface, which makes it easier to use for unexperienced users; however, it still requires sufficient knowledge of privacy methods and is also missing the risk assessment functionality. The last evaluated tool, Anonimatron, based on our research, does not seem to be suitable for healthcare data anonymization. Moreover, we would like to emphasize that some of these tools have not been updated for a significant period of time, which means that they may not be compatible with newer technologies.

#### 2.1.3 Interviews with experts

In addition to the literature review and tool evaluation, we interviewed experts from both the public and the private healthcare sectors in Estonia to understand the current situation with data anonymization and sharing in healthcare organizations. We interviewed several professionals who work with data de-identification and sharing, including doctors of different specialties, nurses, doctor's assistants, data analysts, administrative staff, researchers in healthcare and academic institutions, and startup representatives. The most common points were that data de-identification is mostly a manual process, making it time-consuming and uncertain as to whether the data are properly de-identified. Furthermore, if there is an opportunity to avoid data sharing in the first place, they would take it because they do not want to deal with the risks.

#### 2.1.4 Problem summary

Healthcare data anonymization remains a complex, time-consuming task that typically requires technical expertise and knowledge of data protection regulations such as the GDPR and EHDS in the EU and HIPAA in the US. Existing general data anonymization tools are not suitable for health data because of its unique format, structure, and complexity. These tools require manual configuration and data pre-processing before de-identification. With the enforcement of the EHDS, healthcare providers across the EU must enable secure and compliant data sharing, which is challenging for many organizations because the process of preparing data is often manual and they cannot guarantee that de-identification is done according to data privacy regulations. To overcome the technical challenges and meet privacy regulations, we introduce WiseSpace—a de-identification tool tailored for EHRs and designed to be accessible to non-technical users.

## 3 Proposed de-identification for EHR

### 3.1 General overview of de-identification tool and process

WiseSpace, a tool that securely de-identifies EHRs, supports healthcare standards, such as HL7 FHIR (25) and HL7 CDA (26), and formats, such as JSON and XML. The solution consists of two main modules:

**Module 1: De-identification** detects personal identifiers and quasi-identifiers, assesses data scope and sensitivity, and applies de-identification techniques based on the sharing options selected by the user (e.g., trusted party or public share).

**Module 2: Risk Assessment** estimates the risk of re-identification to support an informed decision about sharing data (27).

**Figure 2:**
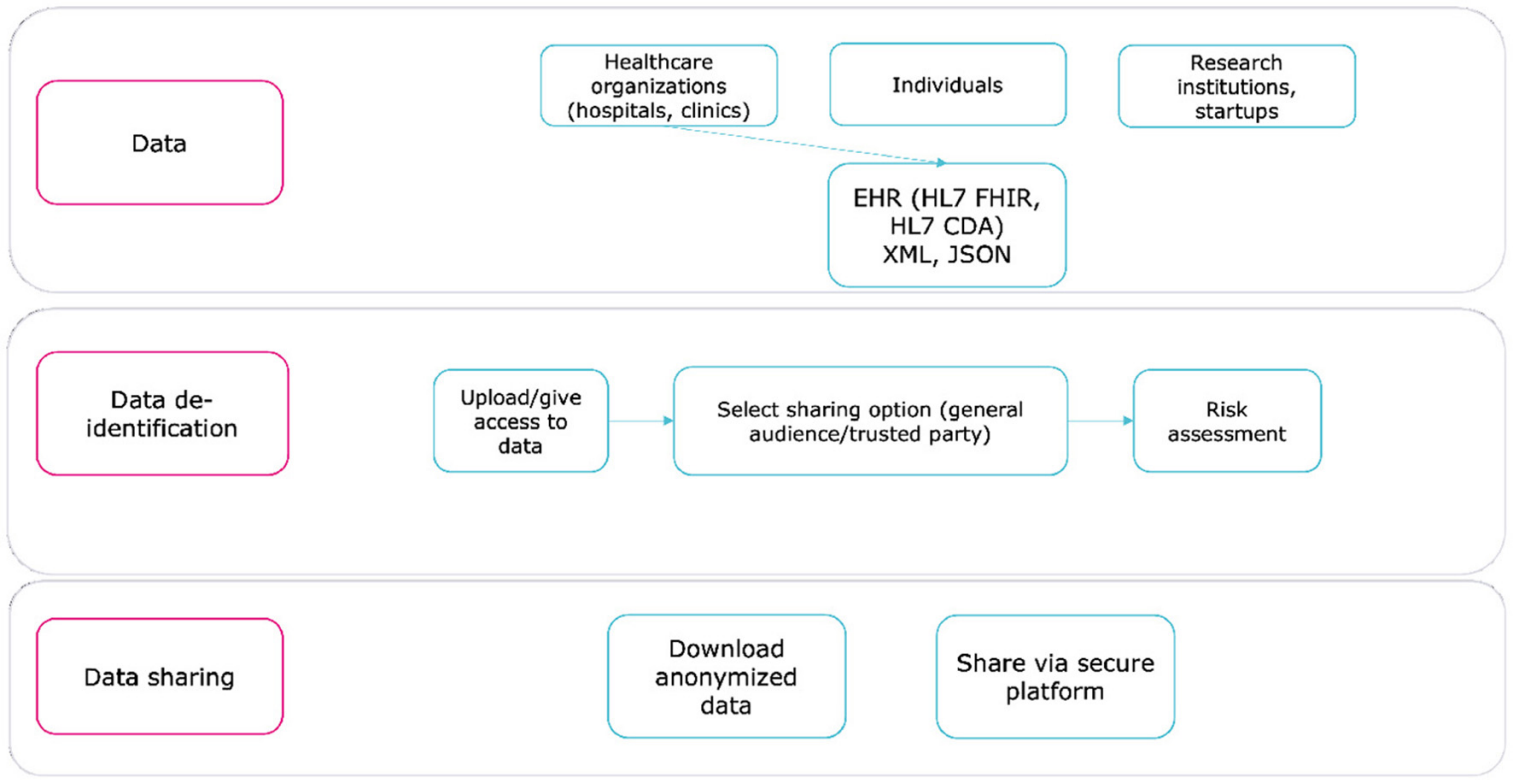
Overview of the WiseSpace de-identification workflow.

The de-identification workflow consists of three steps, illustrated in Figure 2. By automating key processes and minimizing manual input, WiseSpace reduces the likelihood of error and ensures consistency. The tool adapts de-identification rules based on user preferences and dataset characteristics, which makes it suitable for healthcare and research organizations as well as individuals.

### 3.2 Architecture

WiseSpace employs a modular microservice architecture built on Spring Boot, featuring a RESTful API layer with a clear separation of concerns. The API layer utilizes controllers, including a JSON processing controller and an XML processing controller, to manage incoming requests and efficiently route data to suitable processors. Format-specific processing services, such as JSON processors and XML processors, execute customized de-identification workflows tailored precisely to each data format.

The system's de-identification engine implements anonymization and pseudonymization privacy protection techniques. For simpler transformations of individual records, format-specific anonymizers are employed. In addition, the pseudonymization service provides reversible masking for cases where data may require future re-identification.

WiseSpace's processing pipeline incorporates advanced data transformation functionalities. The file upload function initially identifies file types and delegates processing accordingly. Hierarchical data structures are flattened to streamline processing tasks. Privacy protection methods are dynamically selected and applied based on user-defined sharing contexts. Following processing, data reconstruction occurs to rebuild original structures while preserving applied privacy protections. For HL7 resources provided in JSON or XML formats, WiseSpace automatically distinguishes and processes single resources and bundles containing multiple entries, adjusting the privacy protection strategies based on data complexity and sensitivity levels. The risk assessment module evaluates the dataset's characteristics alongside the chosen anonymization parameters, calculating re-identification probabilities and analyzing dataset uniqueness factors.

**Figure 3:**
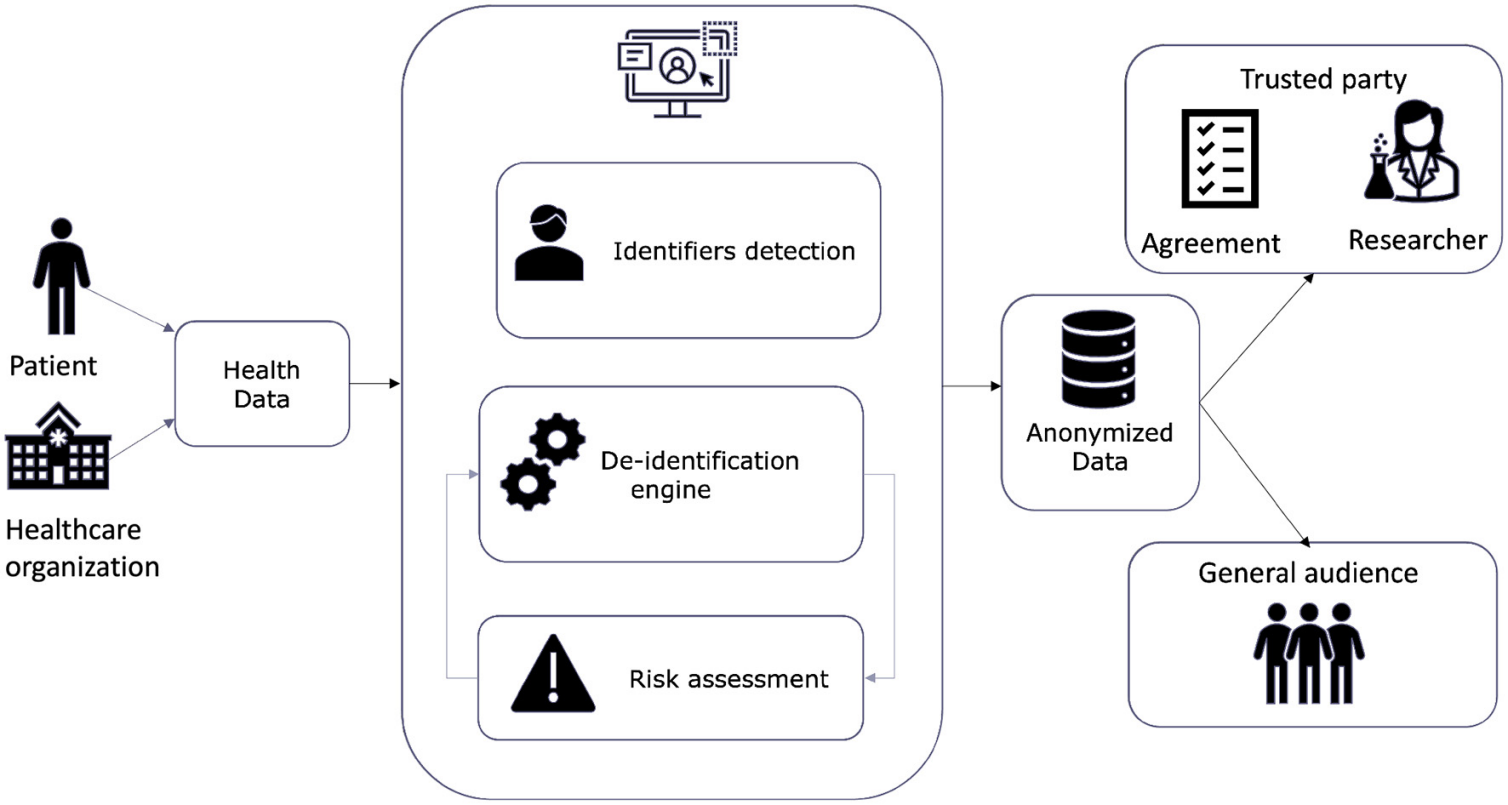
System infrastructure.

Figure 3 illustrates the high-level architecture and data flow of the WiseSpace tool, including input from users (organizations or individuals) and data processing consisting of the detection and categorization of identifiers, the implementation of the de-identification technique, and a re-identification risk assessment. In the final step, de-identified data are shared with a trusted party or a general audience. Sharing with a trusted party (researcher, business) involves party identification and signing an agreement for data use. These services are included as external provider services. In the current version of the solution, we focused on a web-based data de-identification solution. We discuss the possibilities for further development in following chapters.

**Figure 4:**
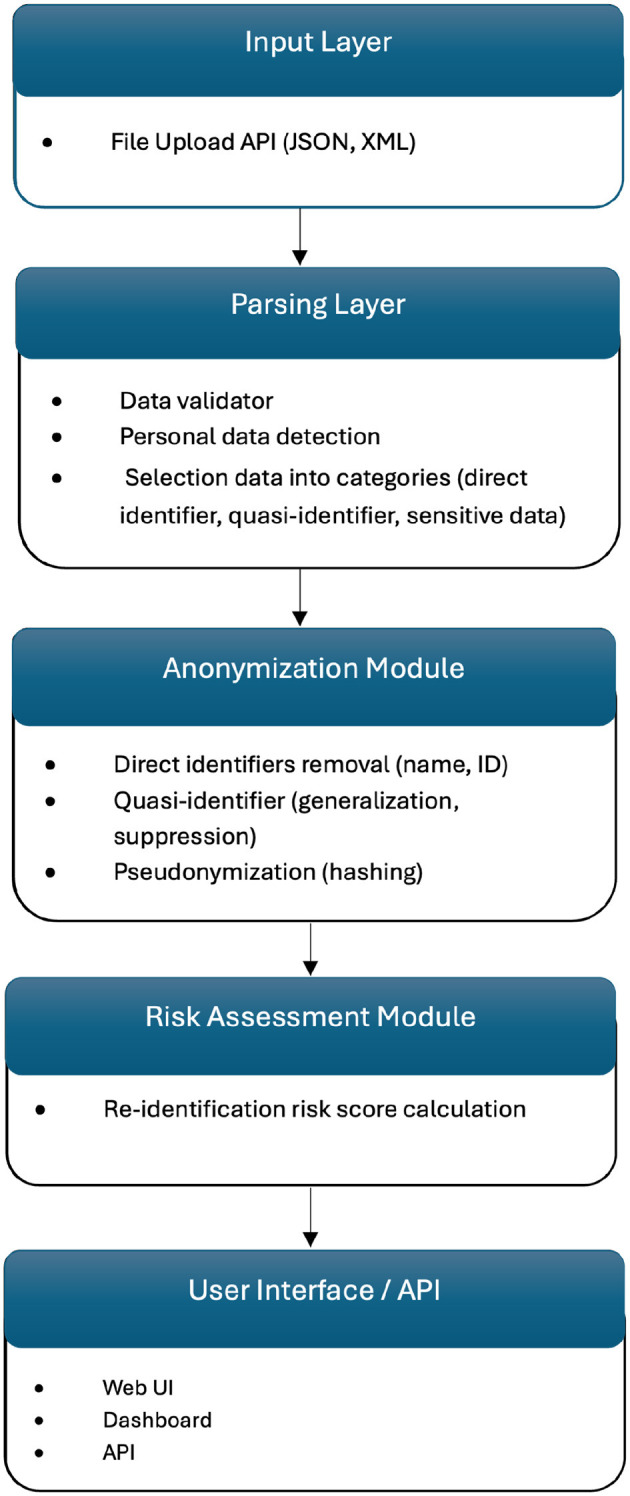
Modular architecture of de-identification tool.

Figure 4 shows the internal technical structure of the solution, highlighting the modular and scalable design.

### 3.3 Technical overview

#### 3.3.1 Requirements

The core functional requirements focus on delivering reliable, healthcare-specific EHR de-identification capabilities. These functional requirements include:

- FR-1. Identifier detection. Automatically detect direct identifiers (e.g., patient names and ID numbers), quasi-identifiers (e.g., dates of birth or zip codes), and sensitive information (e.g., diagnosis) in structured healthcare datasets.
- FR-2. Data de-identification engine. Provide robust anonymization and pseudonymization techniques tailored to different sharing contexts (e.g., trusted partners or public release).
- FR-3. Risk assessment module. Calculate re-identification risks, divide into three categories (low, medium, and high), support users in making informed decisions about data sharing, and propose settings changes and re-calculation if risk of sharing is too high.
- FR-4. Healthcare format support. Ensure compatibility with healthcare standards and data formats such as HL7 FHIR (JSON) and HL7 CDA (XML).
- FR-5. User configuration and automation. Provide an intuitive interface that allows users with common level computer proficiency to perform data de-identification with minimal manual configuration.

Non-functional requirements ensure that the tool operates securely, reliably, and in alignment with healthcare regulations. These include:

- NFR-1. Security. Implement proper protection for data in transit and secure user authentication and access control.
- NFR-2. Regulatory compliance. Adhere to data privacy regulations, including the GDPR and EHDS requirements.
- NFR-3. Usability. Provide a user-friendly interface accessible to healthcare professionals without a technical background.
- NFR-4. Reliability. Guarantee consistent and predictable behavior in the de-identification process.

These requirements are not exhaustive and can be extended as new needs and regulations emerge. For example, future development may include compliance with HIPAA by integrating Safe Harbor de-identification criteria. Enhanced functionality, such as identifying personal data in unstructured free-text fields using natural language processing techniques, could further broaden the tool's applicability. Both functional and non-functional requirements can be refined and divided into more specific subcategories to support evolving healthcare data use cases and ensure robust, adaptable system design.

#### 3.3.2 UI and data flow

**Figure 5:**
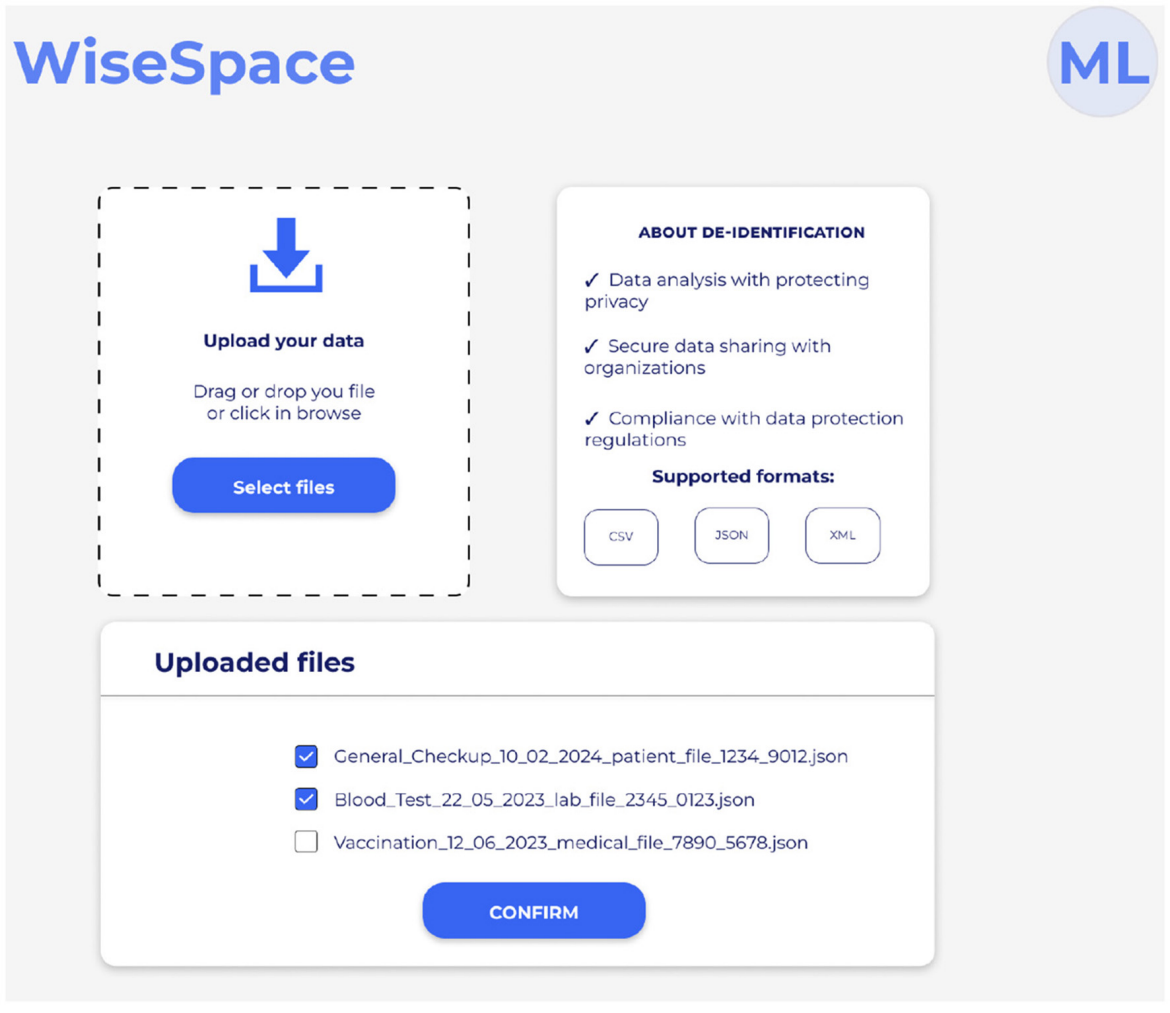
User interface for dataset upload.

**Figure 6:**
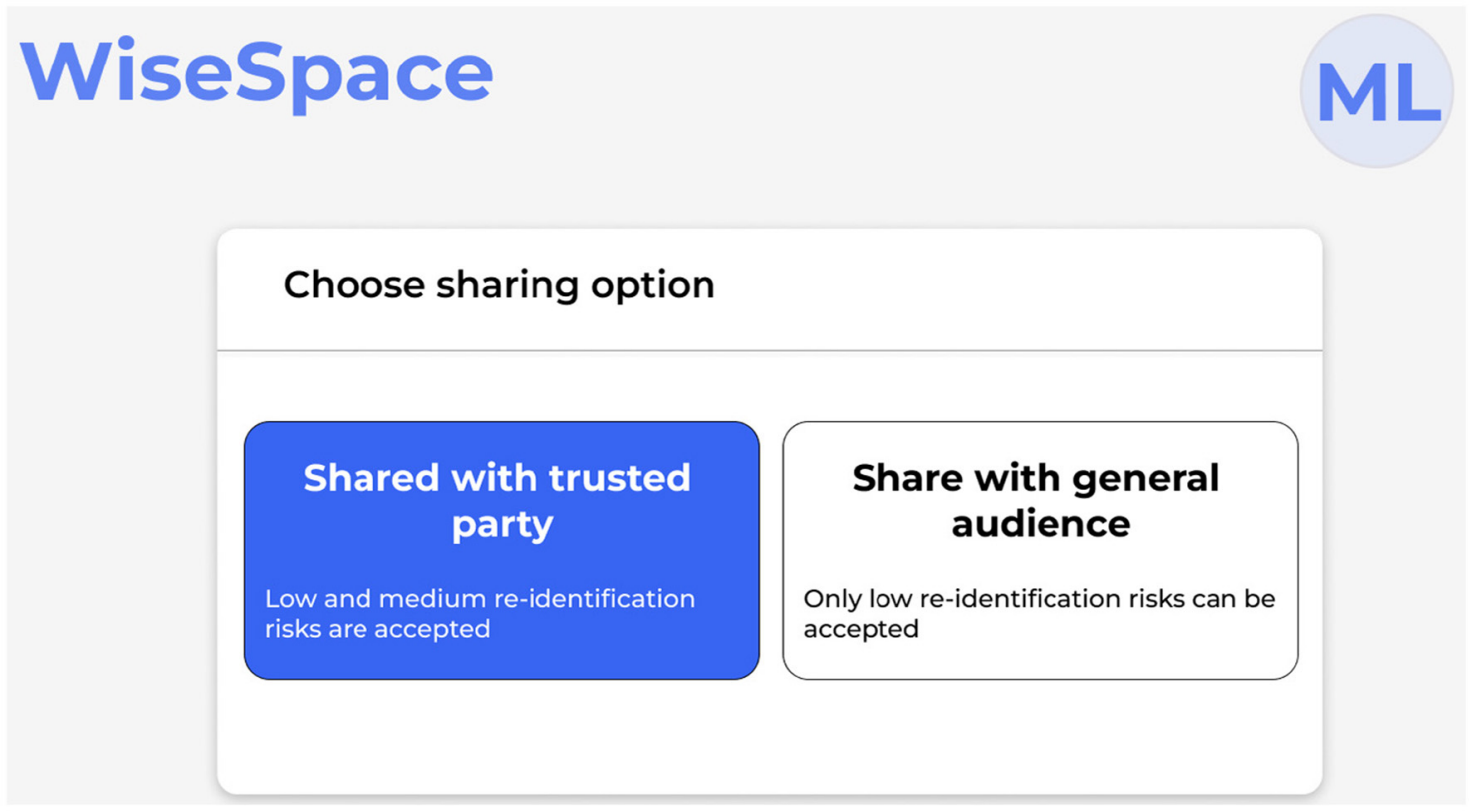
User interface for sharing option selection for de-identified data.

**Figure 7:**
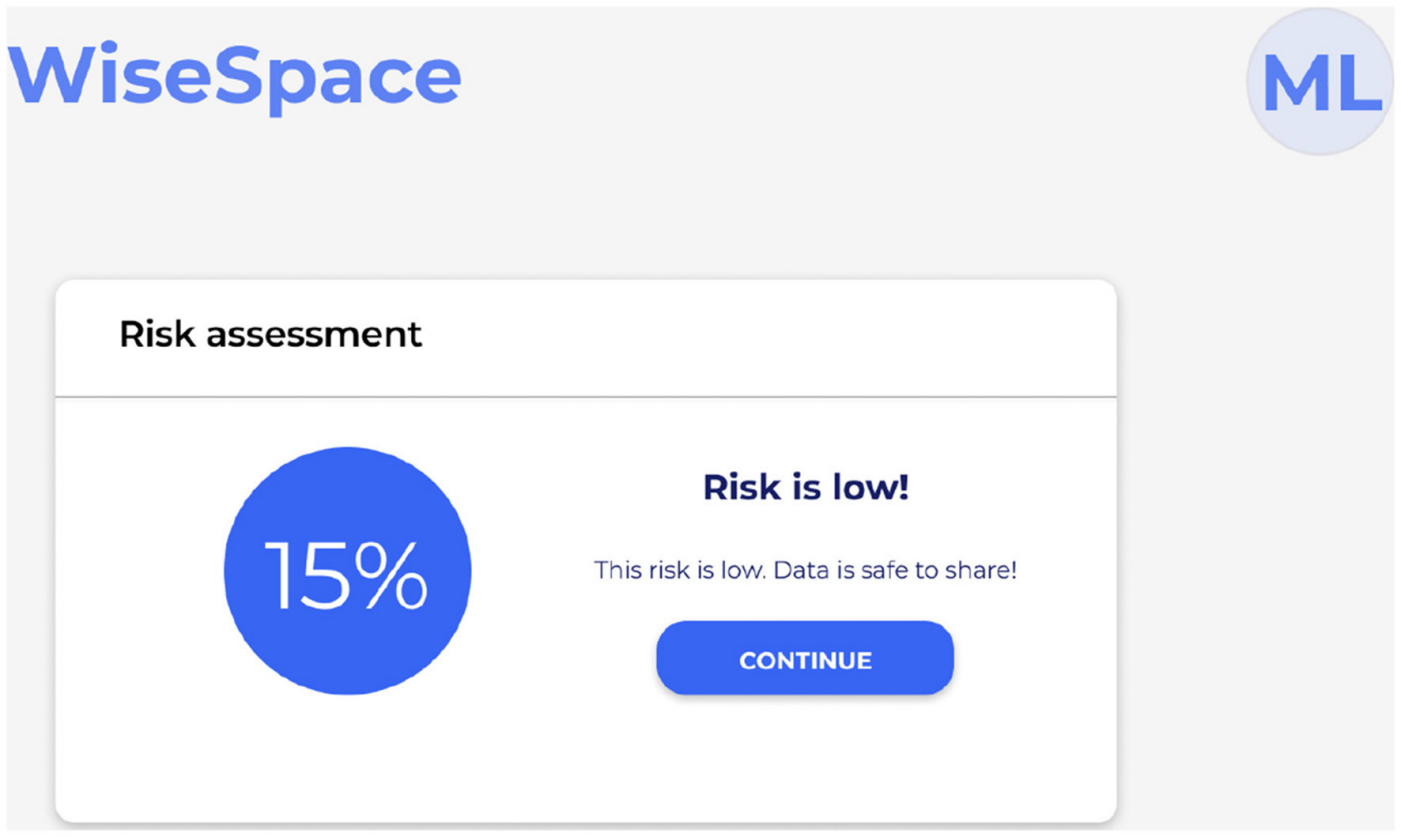
User interface for re-identification risk assessment summary.

UI is designed to be intuitive, user-friendly, and accessible to users despite their technical skill level. Recognizing that many users, including clinicians, administrators, and researchers, may not have advanced technical skills, the UI focuses on simplicity, clarity, and guidance throughout the de-identification process. Users can perform data de-identification following a three-step guided process 2: (1) data upload; (2) selection of sharing options; and (3) risk assessment. Figure 5 shows the UI for uploading data, Figure 6 for selecting a sharing option, and Figure 7 for performing a risk assessment (27).

**Step 1**. Upload data. Before uploading data to the system, the user is asked to confirm the necessary legal basis for data processing. As shown in Figure 5, users select the dataset they wish to de-identify (supporting JSON and XML formats). The user can review uploaded files and de-select, if necessary, and confirm.

**Step 2**. Select sharing option. The UI presents two options: sharing with a trusted party; and sharing with a general audience, as shown in Figure 6. Based on this selection and data scope, de-identification techniques are applied. A trusted party could be an external partner, an individual (e.g., researcher or student), or an organization (e.g., startup, pharmaceutical company, or other). To receive trusted party status, the partner signs an agreement with the healthcare organization on sharing data. The agreement includes rules of data use, limitations, and restrictions (e.g., prohibited use for re-identification attempts, commercial purposes, or sharing with other parties) and liability. Sharing with a general audience implies the possibility for public release. This means that once data are released, there is no control over the receiving party, which may entail a higher risk for re-identification attempts or misuse. To protect personal data, only datasets with a low re-identification risk can be accepted.

**Step 3**. Run risk assessment. The system automatically evaluates the risk of re-identification and provides a visual summary to support informed decisions. Figure 7 shows a visual presentation of the calculated risk. When sharing with a trusted party, low and medium re-identification risks are accepted. When sharing with a general audience, only a low re-identification risk can be accepted. If the calculated risk appears higher than the threshold, the tool offers the user the opportunity to modify the initial dataset or sharing options to lower the potential risks.

## 4 Evaluation of the proposed solution

### 4.1 Practical experiments

We conducted practical experiments to validate the proposed solution. Due to the sensitive nature of healthcare data, we tested our solution with Synthea open-source synthetic patient data (28). Synthetic data are designed to realistically simulate patient data but do not contain actual personal information, making its use free of risk and privacy and security restrictions for testing purposes. For research purposes, we used generated datasets in the HL7 FHIR and HL7 CDA formats. We performed testing on individual records and EHR dataset.

### 4.2 Use cases

WiseSpace is designed for a diverse range of users and data-sharing scenarios. More specifically, it is suitable for organizations and individuals, for integration into information systems, or as a standalone web-based solution.

#### 4.2.1 For healthcare organizations

Healthcare organizations, such as hospitals and clinics, routinely manage sensitive patient data stored in EHRs. Secondary data use, such as for research, quality improvement, and collaboration with external partners, requires careful consideration of privacy, security, and legal compliance. However, the complexity of healthcare data formats combined with the requirement to meet data protection and sharing regulations (GDPR and EHDS) pose significant challenges for secure and compliant data sharing. General-purpose anonymization tools often lack support for healthcare-specific formats and require advanced technical knowledge for effective application. On the other hand, manual de-identification processes are time-consuming, ripe for errors, and costly.

The WiseSpace de-identification tool offers a comprehensive solution tailored to the needs of healthcare organizations. The tool can be integrated into existing healthcare information systems or utilized as a standalone platform, providing an automated, user-friendly interface for the de-identification of structured EHR.

Healthcare professionals, data analysts, and administrative staff can use the tool without specialized technical skills. In addition, healthcare organizations can use the platform for de-identified data sharing with trusted parties, including filtering functionality, agreement management, verification of third party identities, and defining rules and restrictions for data sharing.

#### 4.2.2 For individuals

Under the GDPR, individuals possess the right to access their personal health data and request it in a machine-readable format, such as JSON or XML. Exercising this right, patients can obtain a structured EHR from their healthcare providers and utilize it for personal purposes. However, sharing personal health information with third parties or for AI analysis raises significant privacy concerns. To address this challenge, the WiseSpace de-identification tool empowers individual users to securely de-identify their personal health records, ensuring their privacy is preserved before sharing. One possibility for further use is to contribute the de-identified dataset to research projects. Another possibility is to utilize anonymized health data in AI-driven applications, such as personal health analytics or decision support systems, without exposing sensitive personal information.

#### 4.2.3 For research organizations

Research organizations, including academic institutions, clinical research centers, and pharmaceutical companies, require access to high-quality health data for studies and healthcare innovation. WiseSpace provides research organizations with a comprehensive solution for the de-identification of structured health data and serves as a platform for secure data sharing with advanced filtering functionalities. On this platform, data from EHRs, clinical trials, and individuals can be collected. Organizations can use the platform to share de-identified datasets with trusted parties. The platform allows individual users to donate de-identified personal health data and expand research datasets with real but de-identified patient information.

## 5 Analysis and discussion

### 5.1 Related work

As part of our research, we identified available anonymization tools. In Vovk et al. (10), we also identified eight tools that use anonymization methods: ARX, scdMicro, ShinyAnonymizer, μ-ANT, Anonymizer, Amnesia, Anonimatron, and μ-ARGUS. Several tools offer data anonymization; some of them are presented in scientific publications. The paper Prasser et al. (29) introduces ARX—an open-source anonymization tool, including various settings for data de-identification and risk analysis. ARX utilities several anonymization methods such as k-anonymity, l-diversity, t-closeness σ-disclosure privacy, β-likeness, σ-presence, k-map, and (ϵ, σ)-differential privacy (20). The paper (22) introduces μ-ANT – an open source microaggregation-based anonymization tool that uses k-anonymity and t-closeness anonymization methods.

In our research, we discovered that reaching the proper level of data de-identification using an anonymization tool requires technical knowledge and a deep understanding of the field. Users must select data that is considered an identifier, a quasi-identifier, or sensitive data and apply the proper anonymization methods (19). In addition, we would like to point out that anonymization tools are not tailored to healthcare-specific data formats and standards but rather to general data anonymization.

### 5.2 Regulations

#### 5.2.1 GDPR

The GDPR (11), which entered into force on 25 May 2018, forms the foundation of the European Union's data protection framework. It establishes fundamental principles for personal data processing, including lawfulness, fairness, transparency, purpose limitation, data minimization, accuracy, storage limitation, integrity, and accountability. The GDPR classifies health data as “special category” and sets strict conditions for its processing to protect individuals' privacy and rights.

WiseSpace supports users in meeting the GDPR requirements as follows:

- Lawfulness, fairness, and transparency. Data subjects must be informed about anonymization practices—the system provides information about rules and techniques and notifies the user before uploading data, asking them about the legal basis for data processing.
- Purpose limitation. The tool is intended to process data only for legitimate secondary uses, such as research.
- Data minimization. The user can select the EHR that is shared as well as which data from the record are shared. For example, an EHR contains data about vaccinations and surgery but the user can select and share only data about vaccinations.
- Accuracy. The tool ensures that data anonymization does not lead to misleading or inaccurate data and validates anonymization results for data quality.
- Storage limitation. The tool ensures that identified data are not stored longer than necessary. Identifiable data are not stored within the tool and are processed only in the de-identification process.
- Accountability. We are working on documentation showing compliance with the GDPR during the anonymization process and planning to periodically assess the anonymization techniques for robustness.
- Secure data sharing. The tool applies proper cybersecurity and privacy measures through the de-identification cycle.

#### 5.2.2 EHDS

The EHDS, which entered into force on 26 March 2025, aims to establish a common regulatory framework for accessing and sharing electronic health data across the EU (3). The EHDS empowers individuals with greater control over their health data, allowing them to access, control, and share their EHRs for primary use and enable secure reuse for research, innovation, policy-making, and regulatory purposes (secondary use). The EHDS requires the use of secure processing environments and data minimization techniques, including anonymization and pseudonymization, as prerequisites for secondary data access.

WiseSpace supports users in meeting EHDS regulation requirements as follows:

- Support individuals in accessing, controlling, and reusing their EHRs. Utilizing the right to data portability provided by Article 20 of the GDPR, individuals have the right to receive their personal data in a structured, commonly used, and machine-readable format. Furthermore, using the tool, individuals can securely de-identify data and donate it for research or other purposes.
- Data protection by design and by default (Article 25 of the GDPR). Using the de-identification tool ensures that pseudonymization or anonymization happens before data sharing with third parties.
- Secondary use governance. The tool ensures the proper level of de-identification that balances data privacy and utility for secondary data use. Given sensitive nature of EHRs, Recital 72 of the EHDS emphasizes application of data minimization principle and de-identification, specifically pseudonymization and anonymization, at the earliest possible stage in the data sharing process for secondary use. To meet this requirement, controllers, including health data access bodies and health data holders, can employ WiseSpace as the initial step in the data sharing workflow. This approach reduces the exposure of personal data to unauthorized parties and protects patient privacy. Furthermore, when EHR datasets are already de-identified, WiseSpace can be used to perform risk assessment and verify that the privacy protection level is sufficient.
- Anonymization and pseudonymization (EHDS and GDPR). The tool ensures proper and reliable de-identified techniques for EHRs. As specified in Recital 65 of the EHDS, health data access bodies are responsible for applying tested techniques to ensure privacy in the context of secondary data use. These techniques include pseudonymization, anonymization, generalization, and suppression of personal data (3). WiseSpace applies those techniques to simplify dataset preparation for secondary use.
- Restricted access. The tool ensures that only authorized parties have access to data via the data sharing platform or during the de-identification process.
- Secure processing environments. The tool ensures secure data handling through the de-identification cycle and data sharing platform.
- Data quality and interoperability. The tool ensures technical interoperability standards, supports common data formats, and ensures that data remain usable for research purposes without compromising privacy.
- Prohibition of re-identification. The tool includes a feature that notifies users that any attempt to re-identify data is prohibited.

### 5.3 Future work

In the current version, we focused on developing a web-based anonymization tool; however, in the future, it is possible to use an API and a de-identification engine to connect our solution directly to hospital systems. Moreover, we have discussed the development of the platform for data sharing for healthcare and research organizations, where de-identified data can be securely shared with researchers and other trusted parties.

Currently, the solution works on pre-defined rules and identifier detection. However, in the future, the solution can benefit from AI components for better identifier detection and further possibilities to implement de-identification based on free text.

## 6 Conclusion

This study describes research based on DS methodology that aims to develop a treatment to address data sharing and de-identification challenges, enabling the secure, regulatory-compliant de-identification of healthcare data, particularly for non-technical users. We present WiseSpace—a tool designed to align with healthcare data standards and enhance data privacy and security. It supports organizations in meeting the requirements of the GDPR and the EHDS on data sharing. WiseSpace simplifies the path to GDPR and EHDS compliance for diverse stakeholders, including healthcare providers and researchers. It facilitates the secure secondary use of EHRs by unlocking opportunities for innovation and collaboration while protecting patient privacy. The tool is validated using realistic synthetic data and different scenarios, including individual EHR de-identification and EHR dataset de-identification. Furthermore, WiseSpace lays the foundation for future integration into larger healthcare ecosystems, including integration with hospital information systems and connection to healthcare or research institution data sharing platforms.

## Data Availability

The raw data supporting the conclusions of this article will be made available by the authors, without undue reservation.
